# Youth Responses to Social Media Influencers Discussing Mental Health Online

**DOI:** 10.1192/j.eurpsy.2023.1811

**Published:** 2023-07-19

**Authors:** E. Adeane, K. Stasiak

**Affiliations:** 1 School of Psychology; 2 Department of Psychological Medicine, University of Auckland, Auckland, New Zealand

## Abstract

**Introduction:**

Social media “influencers” are popular online users with large audience bases they are paid to advertise products or services to. Influencers have enormous reach and sway with young people, combining the relatability of peers and the prestige of celebrities. Many researchers have investigated the role of influencers in marketing to youth, and there is a growing interest in public health. However, despite young people’s interest and poor outcomes in mental health, very little research has examined the role of influencers in this field. While a very small number of ethnographic observations about influencers discussing mental health have generated some initial insights on the topic, they contain neither the breadth nor depth to consider the potential impacts of these influencers on young people. To understand the phenomenon of influencers discussing mental health, we need a broad description of what is occurring, how the parties involved feel about it, and what possible effects it may be having on young people.

**Objectives:**

The aim of this research was therefore to explore the role of social media influencers in young people’s knowledge, perspectives and behaviours relating to mental health.

**Methods:**

Researchers conducted digital interviews (text, phone or video-chat) with 21 young people (aged 16-24) and 7 local influencers (18 years plus). Influencers had over 5,000 followers, engaged in sponsored content within 6 months and posted at least once a week. Young people had an interest in mental health and followed at least one of the described influencer accounts.

**Results:**

The preliminary findings reveal four key tensions in how participants view the role of influencers in discussing mental health online. Firstly, participants felt it was important for influencers to be neither overly negative or overly positive in representing mental health, by remaining realistic yet recovery-focused. Second, it was suggested influencers should treat the topic of mental health with appropriate reverence, by taking it seriously but still presenting it in a friendly, youth accessible way. Thirdly, participants suggested influencers should consider how often and openly they discuss mental health, finding a balance between repression and over-expression. Finally, participants valued hearing from both personal and professional perspectives on mental health, and suggested the ideal influencer would share both personal experience and scientific evidence.

**Conclusions:**

This exploratory research is the first step in investigating the possible use of social media influencers in mental health promotion and may be of interest to service providers and health promotion agencies considering this marketing strategy. The research also offers recommendations for popular social media users currently engaging in discussions of mental health online for how to discuss mental health in a way that is most likely to be positively received by young people.

**Disclosure of Interest:**

None Declared

